# Real-world evaluation of the Stemoscope electronic tele-auscultation system

**DOI:** 10.1186/s12938-022-01032-4

**Published:** 2022-09-06

**Authors:** Muge Fan, Qiuli Wang, Jiaqi Liu, Lingyun Su, Bingjian Wang, Hai Xu, Qing Li, Zhi Zhang

**Affiliations:** 1 Department of Cardiology, Shanghai General Hospital, Shanghai Jiaotong University School of Medicine (Initially Named “Shanghai First People’s Hospital”), 100 Haining Road, Shanghai, 200080 People’s Republic of China; 2 Department of Nursing, Shanghai General Hospital, Shanghai Jiaotong University School of Medicine (Initially Named “Shanghai First People’s Hospital”), Shanghai, China; 3 Department of Cardiology, Nanjing Medical University Affiliated with Huai’an First People’s Hospital, Huai’an, Jiangsu, China; 4 Department of Critical Care Medicine, Shanghai General Hospital, Shanghai Jiaotong University School of Medicine (Initially Named “Shanghai First People’s Hospital”), Shanghai, China

**Keywords:** Tele-auscultation, Auscultation, Heart murmur, Valvular heart diseases, Electronic stethoscope

## Abstract

**Background:**

With the spread of COVID-19, telemedicine has played an important role, but tele-auscultation is still unavailable in most countries. This study introduces and tests a tele-auscultation system (Stemoscope) and compares the concordance of the Stemoscope with the traditional stethoscope in the evaluation of heart murmurs.

**Methods:**

A total of 57 patients with murmurs were recruited, and echocardiographs were performed. Three cardiologists were asked to correctly categorize heart sounds (both systolic murmur and diastolic murmur) as normal vs. abnormal with both the Stemoscope and a traditional acoustic stethoscope under different conditions. Firstly, we compared the in-person auscultation agreement between Stemoscope and the conventional acoustic stethoscope. Secondly, we compared tele-auscultation (recorded heart sounds) agreement between Stemoscope and acoustic results. Thirdly, we compared both the Stemoscope tele-auscultation results and traditional acoustic stethoscope in-person auscultation results with echocardiography. Finally, ten other cardiologists were asked to complete a qualitative questionnaire to assess their experience using the Stemoscope.

**Results:**

For murmurs detection, the in-person auscultation agreement between Stemoscope and the acoustic stethoscope was 91% (*p* = 0.67). The agreement between Stemoscope tele-auscultation and the acoustic stethoscope in-person auscultation was 90% (*p* = 0.32). When using the echocardiographic findings as the reference, the agreement between Stemoscope (tele-auscultation) and the acoustic stethoscope (in-person auscultation) was 89% vs. 86% (*p* = 1.00). The system evaluated by ten cardiologists is considered easy to use, and most of them would consider using it in a telemedical setting.

**Conclusion:**

In-person auscultation and tele-auscultation by the Stemoscope are in good agreement with manual acoustic auscultation. The Stemoscope is a helpful heart murmur screening tool at a distance and can be used in telemedicine.

## Background

The first stethoscope was invented by Laennec in 1816 and remains a commonly used tool today [1]. Till now, auscultation remains a vital component of the initial cardiopulmonary examination. A heart murmur caused by valvular heart disease (VHD) is the most common reason for referral to a cardiologist. Since 1956, various electronic stethoscopes have been developed to capture heart sounds and save them in digital format. Today, electronic stethoscopes have a variety of sizes and shapes [2–4]. An excellent electronic stethoscope should have the ability to both eliminate background noise and amplify heart sounds. The 3M Littmann electronic stethoscope (3200) can store heart sounds and transmit them to the computer via Bluetooth. The Stemoscope we used was wireless and could store and share heart sounds more easily than most electronic stethoscopes. [5] These electronic stethoscopes also help teach medical students more effectively [6].

Some studies have compared acoustic and electronic stethoscopes in clinical practice [2]. Kalinauskien E et al. showed that there is an indication of increased sensitivity when using the electronic stethoscope in obese patients [7]. Fontaine E et al. suggested that in contrast to the Littmann Cardiology III, the electronic Littmann 3200 can help clinicians to better auscultate cardiac and respiratory areas during air medical transport in a Falcon fixed-wing aircraft [8].

Tele-auscultation has become more critical in the past few years, especially with the emergence of COVID-19 [9, 10]. There has been increased interest in the development of tele-auscultation systems to screen for heart and lung diseases [11]. We have previously used an electronic stethoscope (Stemoscope) (Hulu Devices) for tele-auscultation in the containment capsule at Leishenshan Hospital, Wuhan, China [5]. Park DE et al. found that conventional auscultation and remotely classified digital auscultation displayed moderate concordance for the presence/absence of wheezing and crackles among children aged 1–59 months enrolled in the Pneumonia Etiology Research for Child Health (PERCH) case–control study [12]. Behere et al. suggested that an Eko Core system may be a useful screening tool to help identify patients at a distance who would benefit from a further cardiac assessment [13]. Wu et al. developed an electronic stethoscope and a classification algorithm for cardiopulmonary sounds [14]. Many wearable stethoscopes for long-term ambulatory respiratory and cardiac health monitoring have been created [15–17]. Moreover, artificial intelligence (AI) algorithms have been increasingly used recently and have been applied in automatic auscultation [18–21].

Despite improvements in technology, electronic stethoscopes have not been widely adopted in the daily practice of telemedicine. The reason for the low rate of usage is that there is no product that can transmit and store auscultation information over long distances in an accurate, reliable, and user-friendly manner. In addition, many cardiologists still believe that the quality of tele-auscultation is inferior to that of in-person auscultation.

Although tele-auscultation has existed for a long time, remote auscultation has not featured much in telemedicine. In addition to the above concerns about the quality of tele-auscultation and users' habits, one of the other main reasons might be that there is a lack of products that are portable, accessible, and can easily access the internet. Since the Stemoscope was proven to be a successful electronic stethoscope worldwide in the past few years, we planned to test its tele-auscultatory function in this study.

Studies comparing acoustic and electronic stethoscopes in person and tele-auscultation are scarce. In this study, the Stemoscope and a traditional acoustic stethoscope were compared for patients with abnormal heart sounds, including heart murmurs, and other pathologic findings. Both in-person and tele-auscultation (recorded heart sounds) were compared to test the hypothesis that digitally recorded heart findings supply equally sufficient information as in-person auscultation for clinical decisions.

## Results

Twenty-five men and 32 women were enrolled in this study (Table 1). The mean age was 61.5 (4–84) years. Four patients were excluded from the study due to the poor quality of the recording, most of whom had extremely low auscultation even when using mechanical stethoscopes. Three patients were excluded from the study due to the absence of echocardiography. In total, the number of individual recordings reached 285, with 254 valid recordings from 57 patients, each with auscultations of 5 valves. Echocardiography findings showed that the most frequent valve abnormalities were mitral regurgitation (35.85%), tricuspid regurgitation (14.15%), and aortic regurgitation (11.32%) (Table 2).

**Table 1:** Baseline characteristics of patients

Variable	Value
Age, mean ± SD, years	61.58 ± 18.30
Male, n (%)	25 (43.86%)
*Echocardiography findings (%)*	
Aortic regurgitation	12 (11.32%)
Mitral regurgitation	38 (35.85%)
Mitral valve prolapse	3 (2.83%)
Tricuspid regurgitation	15 (14.15%)
Aortic stenosis	9 (8.49%)
Mitral stenosis	4 (3.77%)
Ventricular septal defect	3 (2.83%)
Atrial septal defect	1 (0.94%)
Mitral valve replacement	14 (13.21%)
Tricuspid valve replacement	2 (1.89%)
Aortic valve replacement	5 (4.72%)

**Table 2:** Agreement of in-person auscultation results between the Stemoscope and an acoustic stethoscope among three cardiologists

	Cardiologist 1		Cardiologist 2		Cardiologist 3	
	Acoustic		Acoustic		Acoustic	
	Abnormal	Normal	Abnormal	Normal	Abnormal	Normal
*Stemoscope*						
Abnormal	150	9	148	11	144	15
Normal	9	90	15	84	13	86
Agreement	93%		90%		89%	
P value	1		0.44		0.57	
Kappa value	0.85		0.78		0.78	

**Fig. 1:**
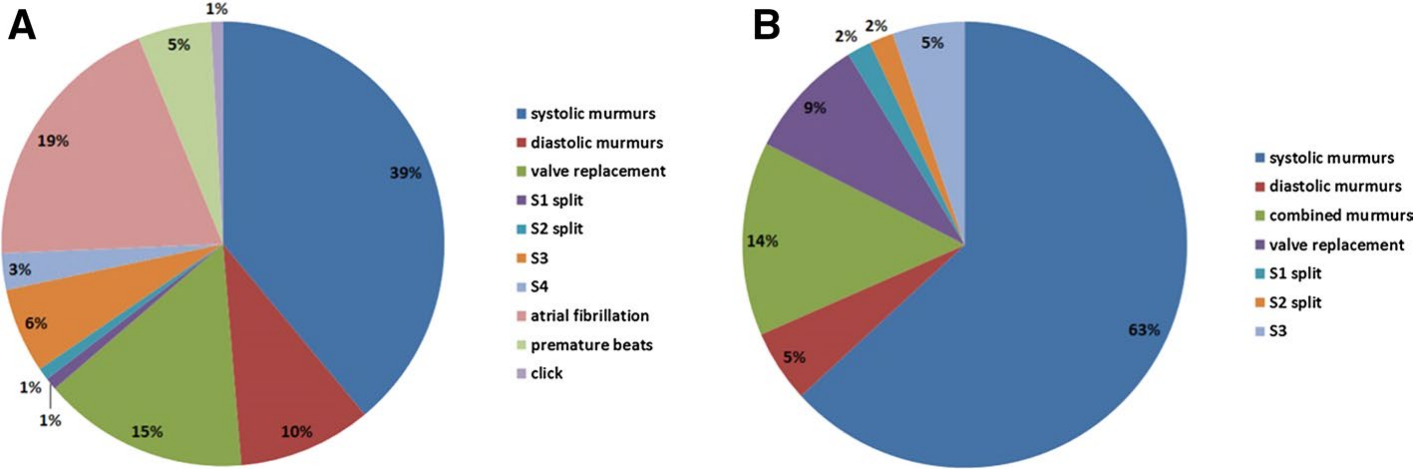
Distribution of in-person auscultation findings. **A** Total abnormal auscultatory findings including heart arrhythmias. **B** Abnormal in-person auscultatory findings

The distribution of in-person auscultation findings by cardiologist 1 is detailed in Fig. 1. Thirty-six (63%) patients had an isolated systolic murmur (systolic ejection murmur and systolic regurgitation murmurs). Three (5%) patients had an isolated diastolic murmur (mitral stenosis murmur and aortic regurgitation murmurs). Eight (14%) patients had combined murmurs. Among them, 11 patients had a secondary sound in addition to a murmur, such as a split S1 (1), a fixed split S2 (1), S3 (third heart sound) (3), a prosthetic valve sound (5), and a click (1). Six of the patients had premature beats, and 22 had atrial fibrillation.

The analysis of comparisons between the Stemoscope and the acoustic stethoscope in-person auscultation is detailed in Table 3. Compared with the mechanical stethoscope, the Stemoscope allowed cardiologists to correctly categorize sounds (both systolic murmur and diastolic murmur) as normal vs. abnormal with an agreement of 91% (89–93%) (*p* = 0.67) and a Kappa value of 0.80 (substantial). The total number of patients with heart murmurs was 47, and there were 235 entire recordings (one recording may yield multiple murmurs, i.e., a patient could have both a systolic murmur and a diastolic murmur). There were 218 valid recordings, and 17 vague recordings were excluded from the analysis. We also compared the agreement of detecting additional sounds, such as prosthetic valve sounds S3 and S4 (fourth heart sound), which showed no difference (*p* > 0.05).

**Table 3:** Agreement of in-person auscultation and Stemoscope tele-auscultation results among three cardiologists

		Cardiologist 1				Cardiologist 2		Cardiologist 3	
		In-person				In-person		In-person	
		Abnormal		Normal		Abnormal	Normal	Abnormal	Normal
*Tele-auscultation*									
Abnormal		153		14		150	16	141	18
Normal		6		85		12	80	11	88
Agreement		92%				89%		89%	
P value		0.27				0.57		0.26	
Kappa value		0.77				0.77		0.83	

Comparisons between the acoustic stethoscope in-person auscultation and Stemoscope tele-auscultation are detailed in Table 4. Compared with in-person auscultation, the Stemoscope tele-auscultation allowed cardiologists to correctly categorize sounds as normal vs. abnormal with an agreement of 90% (89–92%), (*p* = 0.32) and a Kappa value of 0.79 (substantial). We also compared the agreement of detecting additional sounds, such as prosthetic valve sounds, S3, and S4, which showed no difference (*p* > 0.05).

**Table 4:** Comparisons between echocardiographic findings and Stemoscope tele-auscultation/acoustic in-person auscultation findings (Stemoscope tele-auscultation results of three cardiologists were combined)

	Stemoscope tele-auscultation vs. acoustic in-person auscultation			
	Systolic murmurs		Diastolic murmurs	
	Abnormal	Normal	Abnormal	Normal
*Echocardiography*				
Abnormal	50 vs. 48	3 vs. 4	7 vs. 6	5 vs. 5
Normal	3 vs. 4	0 vs. 0	3 vs. 4	0 vs. 0
Agreement	89% vs. 86%		47% vs. 40%	
Sensitivity	94% vs. 92%		58% vs. 55%	

The analysis of comparisons between echocardiographic findings and Stemoscope tele-auscultation/acoustic in-person auscultation is detailed in Table 5. Compared with echocardiography categorization as normal vs. abnormal, Stemoscope tele-auscultation vs. acoustic in-person auscultation allowed cardiologists (we combined the auscultation results of three cardiologists) to correctly categorize systolic and diastolic murmurs with an agreement of 89% vs. 86% (*p* = 1.00) and 47% vs. 40% (*p* = 0.72), respectively, and with a sensitivity of 94% vs. 92% and 58% vs. 55%, respectively. Three aortic regurgitation murmurs and 1 mitral stenosis murmur were correctly heard with Stemoscope tele-auscultation, but missed with acoustic in-person auscultation.

**Table 5:** Summarized results of different electronic stethoscopes

References	Product name	Tele-auscultation	Smartphone app	Agreement with echocardiography	Comparison with acoustic stethoscope
Our study	Stemoscope	Y	Y	89% for systolic, 58% for diastolic murmurs	90.6%
Kalinauskiene E	3 M Littmann 3200	NA	NA	64.17% by cardiologists, 65% by residents	NA
Chowdhury MEH	Eko Core Digital Stethoscope	Y	Y	NA	87.6%
Ghanayim T	VoqX	NM	NM	86% for aortic stenosis	NA
Hirosawa T	JPES-01	NA	NA	NA	83.3% (vs. stimulator)
Belmont JM	ATI	Y	NA	Kappa = 0.55	88%

NA: not available; NM: not mentioned; Y: yes

**Fig. 2:**
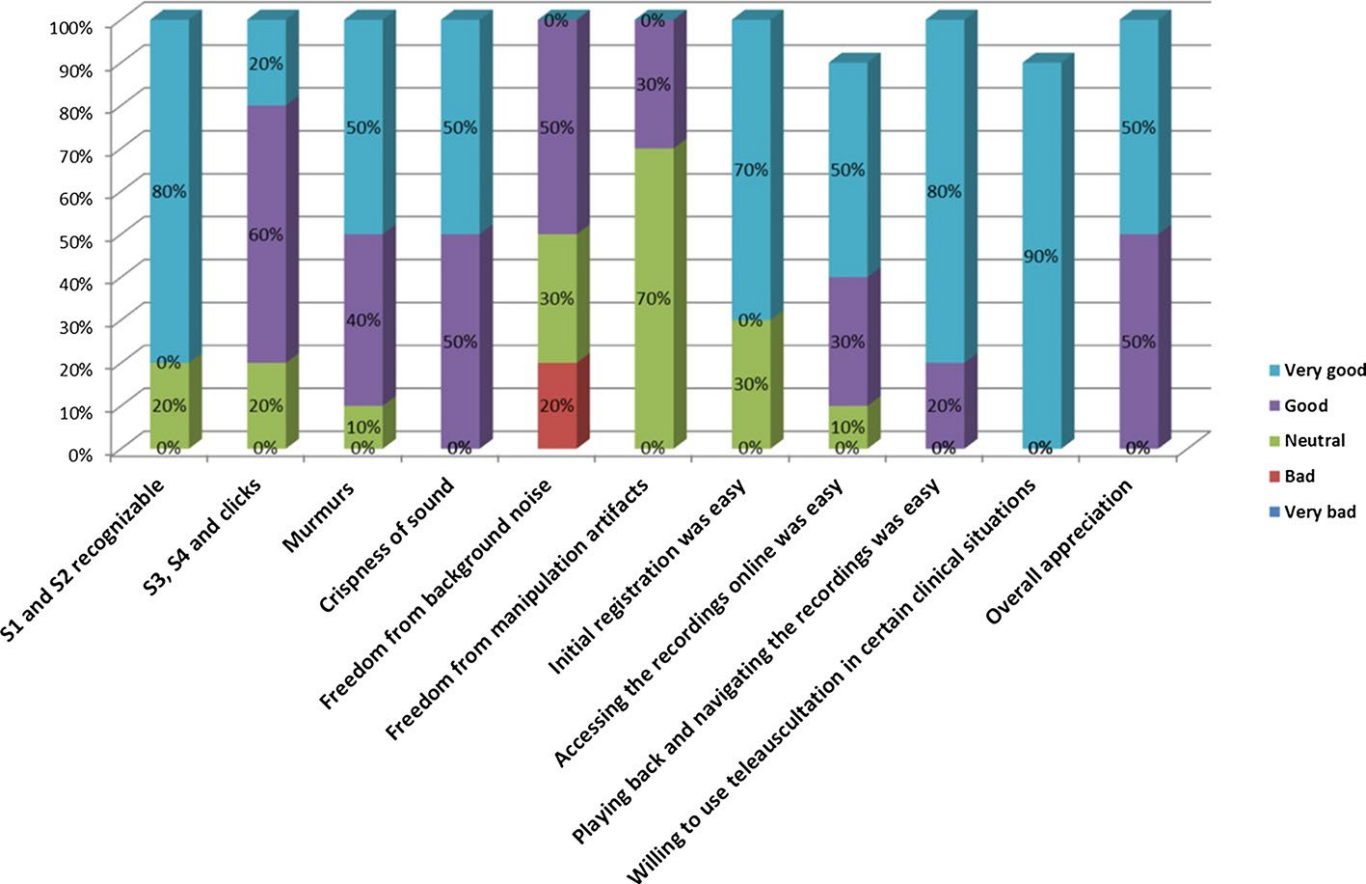
Results of the qualitative survey concerning the experience of using the Stemoscope

Overall, it was very easy to register for an account and use the Stemoscope to listen and share sounds. Additionally, with the phonocardiogram associated with the recordings, the cardiologists could easily adjust the quality of the recording, and it was beneficial when they had difficulties detecting atrial fibrillation and premature beats. Although there were usually subtle differences between sounds heard by acoustic stethoscopes compared to electronic stethoscopes, the sounds recorded by the Stemoscope were much closer to the sounds in real life than those recorded by other brands of electronic stethoscopes. Most frequently, readers felt that the recording imparted a louder, mechanical quality to S1/S2, they needed time to adapt. A qualitative survey of ten cardiologists is detailed in Fig. 2, which shows the feedback on the electronic Stemoscope by in-person and tele-auscultation. Overall, the feedback was positive, with physicians scoring the user experience and the quality of the Stemoscope sounds as good to very good. Nine out of ten users felt the Stemoscope was easy to use and would consider using it in specific clinical settings.

## Discussion

This study introduces a wireless stethoscope that can be used in tele-auscultation. It is tiny, portable, and can be used in containment capsules. For in-person auscultation, there was no difference between the accuracy of the Stemoscope and that of the traditional acoustic stethoscope. For tele-auscultation, there was no difference between the accuracy of the Stemoscope and that of the conventional acoustic stethoscope. When using echocardiography as the reference to clarify valvular lesions, the accuracy and sensitivity of the Stemoscope were similar to those of in-person auscultation by the conventional acoustic stethoscope.

There are few studies on the application of electronic stethoscope systems in long-distance auscultation. Table 5 shows a pooled review of some electronic stethoscopes that are available on the market [7, 10, 13, 18, 22]. A few studies have attempted to compare the results of tele-auscultation systems with those of in-person auscultation, usually by playing recordings to a group of experts to assess reliability and validity [13, 23–26]. The sensitivity and specificity of these studies ranged from 87–100% and 82–98%, respectively. There were no significant differences in accuracy between the two types of stethoscopes (the traditional acoustic stethoscope and Stemoscope) for the cardiologists in both scenarios (in-person auscultation and tele-auscultation); the accuracy was 91% and 90%, respectively. However, there was a trend in which Stemoscope tele-auscultation was more likely to detect S3 and S4 than acoustic stethoscope in-person auscultation, but was less likely to recognize prosthetic valve replacement sounds. This trend may be partly due to the playback function during tele-auscultation, which makes it possible to hear heart sounds in more detail. Prosthetic valve sounds are particularly easy to identify when using a traditional acoustic stethoscope because prosthetic valvular sounds are usually louder than normal S1 and S2 sounds. Since the electronic stethoscope amplifies both prosthetic valvular sounds and ordinary heart sounds, which reduces the sound volume gap between them, it makes it slightly difficult to distinguish them. In addition, we found that the detection rates of atrial fibrillation and premature beats were the same between in-person auscultation and tele-auscultation when calculated in combined valves. Still, the detection rate of premature beats was reduced when we calculated separated valves. This may be because the recording time frame was only 15 s, so if the gap between two premature beats was greater than 15 s, tele-auscultation might fail to detect it. However, persistent atrial fibrillation and frequent premature beats would not be missed. In the future, as real-time tele-auscultation is adopted, there will be no limit of 15 s for recording, so the detection rate of premature beats will be similar to that of in-person auscultation.

There was no significant difference in the accuracy and sensitivity between the electronic Stemoscope and traditional acoustic stethoscopes when using echocardiography as the gold standard, which was similar to other studies [27, 28]. There seemed to be a trend that the Stemoscope was more accurate than acoustic stethoscopes for both systolic (89% vs. 86%) and diastolic (47% vs. 40%) murmurs. We expect that Stemoscope tele-auscultation will be used primarily for screening purposes where sensitivity is paramount. Regardless, the auscultation results should be placed in an appropriate clinical context, ultimately increasing diagnostic accuracy. We also noted that the sensitivity to diastolic murmurs was low, mainly due to the low-frequency characteristic of diastolic murmurs. However, it is also related to the design of this study because the number of diastolic murmurs in this study was relatively small, which led to a significant variation. We also know that excluding murmurs of grade 1 intensity could significantly increase sensitivity, but we did not exclude these [29]. In addition, we enrolled patients with murmurs, so the specificity could not be calculated because there were no patients who did not have murmurs. In our study, the most frequent valvular abnormality was mitral regurgitation, and our cardiologists were aged 40 to 45 years. Because we gradually lose high-frequency hearing as we age [30], electronic stethoscopes may be helpful for geriatric doctors, especially for high-frequency murmurs caused by reflux [31]. Moreover, three aortic regurgitation murmurs and one mitral stenosis murmur were correctly heard with Stemoscope tele-auscultation, but missed with acoustic in-person auscultation, which demonstrated that the electronic stethoscope also has an advantage in recognizing diastolic murmurs.

In this study, echocardiography was used as the gold standard to verify the auscultation results [32, 33]. The echocardiographic clinical report form allows the reporting of all valvular lesions, including stenosis and regurgitation. The main purpose of this study was to confirm heart murmurs rather than to compare diagnostic accuracy with echocardiography, so we did not perform a severity classification of murmurs. Usually, functional murmurs are related to low sound intensity (predominantly grade 1) and early systolic timing. Pathologic murmurs frequently have one or more associated clinical abnormalities [27]. However, the clinical practice's identifying functional or pathological features is sometimes tricky and inaccurate [34–36]. In addition, false-positive auscultation for VHD usually prompts further diagnostic testing, but the accessibility of obtaining an echocardiogram to confirm the diagnosis and initiate disease surveillance varies from country to country. For example, in China, it is very convenient for patients to see a doctor and make an appointment for an echocardiogram; patients sometimes do not even need to make an appointment, which is different from countries where patients are strictly screened to make an appointment for an echocardiogram [35].

A survey of experience using this electronic stethoscope showed that it was easy to use. The Stemoscope can electronically filter, amplify and transfer sounds to a mobile app for storage and sharing. The generated data files can be shared via the web through a smartphone app and can be downloaded or directly played on WhatsApp or WeChat for other users to hear. There is also an option to notify users of live-stream auscultation in real-time. When listening, using earphones is recommended because playing directly with a mobile phone will change the heart sounds and lead to misjudgment. There is an option of a bell or membrane auscultation model with a range of 20–1000 MHz that can be adjusted according to different types of murmurs. It can store and playback patients' heart sounds, which may be helpful for teaching and patient monitoring in addition to tele-auscultation. The Stemoscope allows us to document not only murmurs but also elusive arrhythmias. Two users felt that its noise-free capability needed to be improved. The other comments ranged from good to very good. In particular, the Stemoscope is easier to carry than any other currently available electronic stethoscope. Due to its easy connection with mobile phones, it is convenient to replay and share the files in an app without specialized training.

There are several limitations to our study. The sample size was small; all of the auscultations came from only 57 patients, and only three cardiologists participated in this study. The cardiologists had only two weeks to become familiar with the electronic stethoscope before the study started. The nonrandomized nature of the study, which included only patients who had murmurs, might be a source of selection bias. We also did not compare the intensity of murmurs with the severity of the lesions determined by echocardiography because this study aimed to observe the capability of detecting murmurs by the Stemoscope. Kappa statistics are often used to evaluate consistency, but they require the same number of categories observed by two observers. Since we did not include normal controls without murmurs in the echocardiography-based evaluation, Kappa values and specificity could not be calculated in this comparison.

Regarding the device itself, the iteration of the Stemoscope we studied is designed to be used with headphones rather than with a conventional two-tube stethoscope, which is a limitation but also innovation. Because the cardiologists involved in this study were relatively young, they were ready to accept digital auscultation. Since geriatric cardiologists might have difficulty altering their auscultation habits, we should determine their willingness to adopt electronic stethoscope tele-auscultation.

Finally, many people question the need for auscultation when diagnosing heart disease as other technologies, such as handheld ultrasonic devices, continue to advance. However, the development of the intelligent assisted Stemoscope we are exploring may change their minds.

## Conclusion

The Stemoscope and the traditional stethoscope showed equal accuracy and sensitivity for VHD patients. In addition, the Stemoscope system may be a useful tool to help patients at a distance who need cardiac assessment. There is also an indication that the Stemoscope might be beneficial for older physicians with hearing loss. This hypothesis should be tested in a larger study. Young physicians should also improve their auscultatory skills and be prepared for the future implementation of tele-auscultation.

## Methods

This study (ChiCTR2000038272) aimed to compare the ability of a wireless electronic stethoscope against an acoustic stethoscope in detecting heart murmurs and heart arrhythmias in patients. Fifty-seven patients were included in this study between September 2021 and February 2022. Patients older than one year who was admitted to Shanghai General Hospital were enrolled in a nonrandomized fashion. The institutional ethics board approved this single-center study of the Shanghai General Hospital of Shanghai Jiaotong University. Written informed consent was obtained from all participants. The exclusion criterion was as follows: the investigator believed the patient should not be included (i.e., heart sounds were extremely hard to hear or hard to recognize or were unsuitable for inclusion (patient with absent echocardiography).

**Fig. 3:**
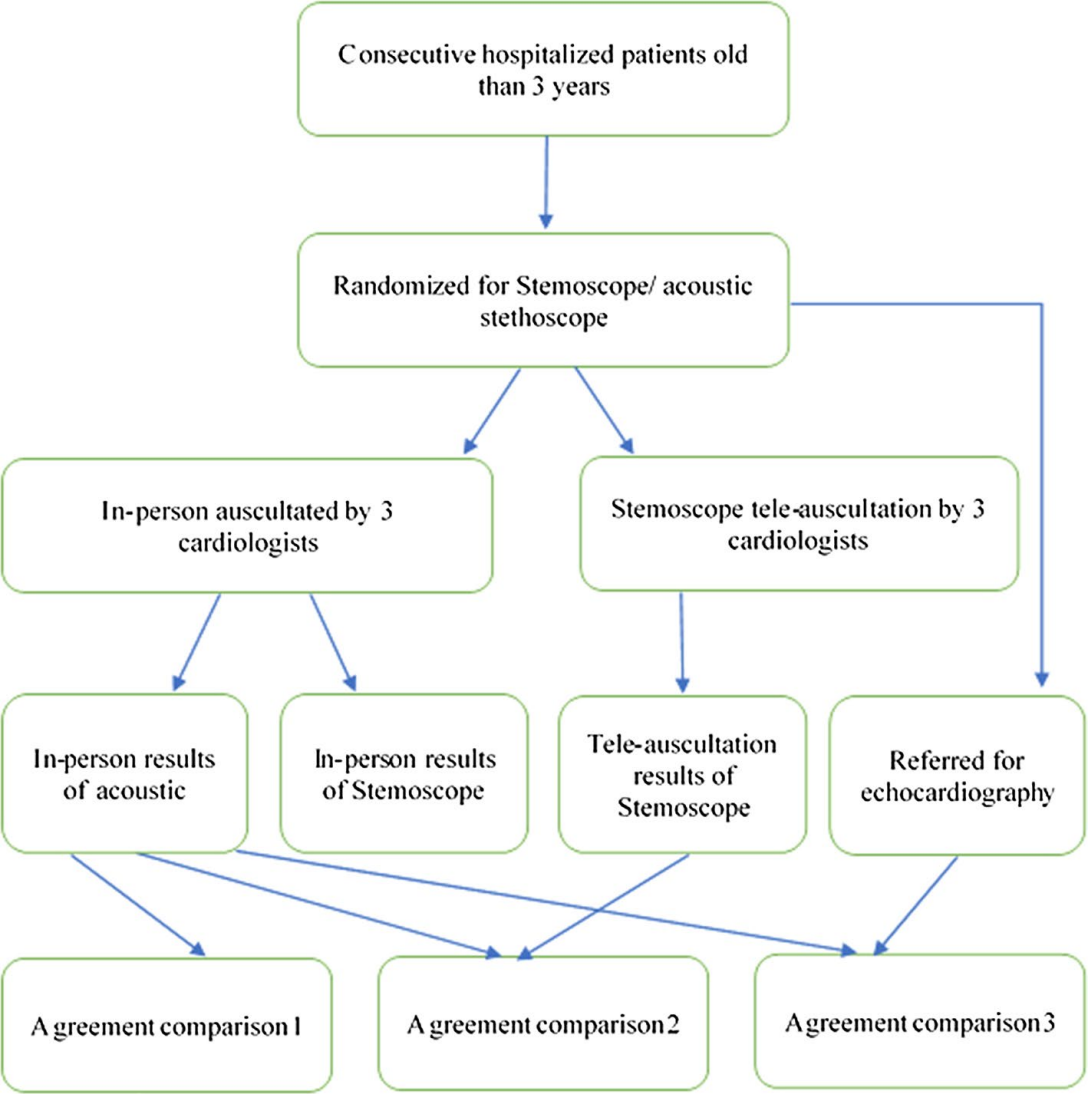
Study flowchart

This study aimed to compare the traditional acoustic stethoscope with an electronic stethoscope in the following three ways: in-person auscultation, tele-auscultation, and echocardiography findings. Fifty-seven patients with abnormal heart sounds were enrolled. Figure 3 shows the flowchart of the study. Patients could be excluded at any step of the study in the case of complications.

**Fig. 4:**
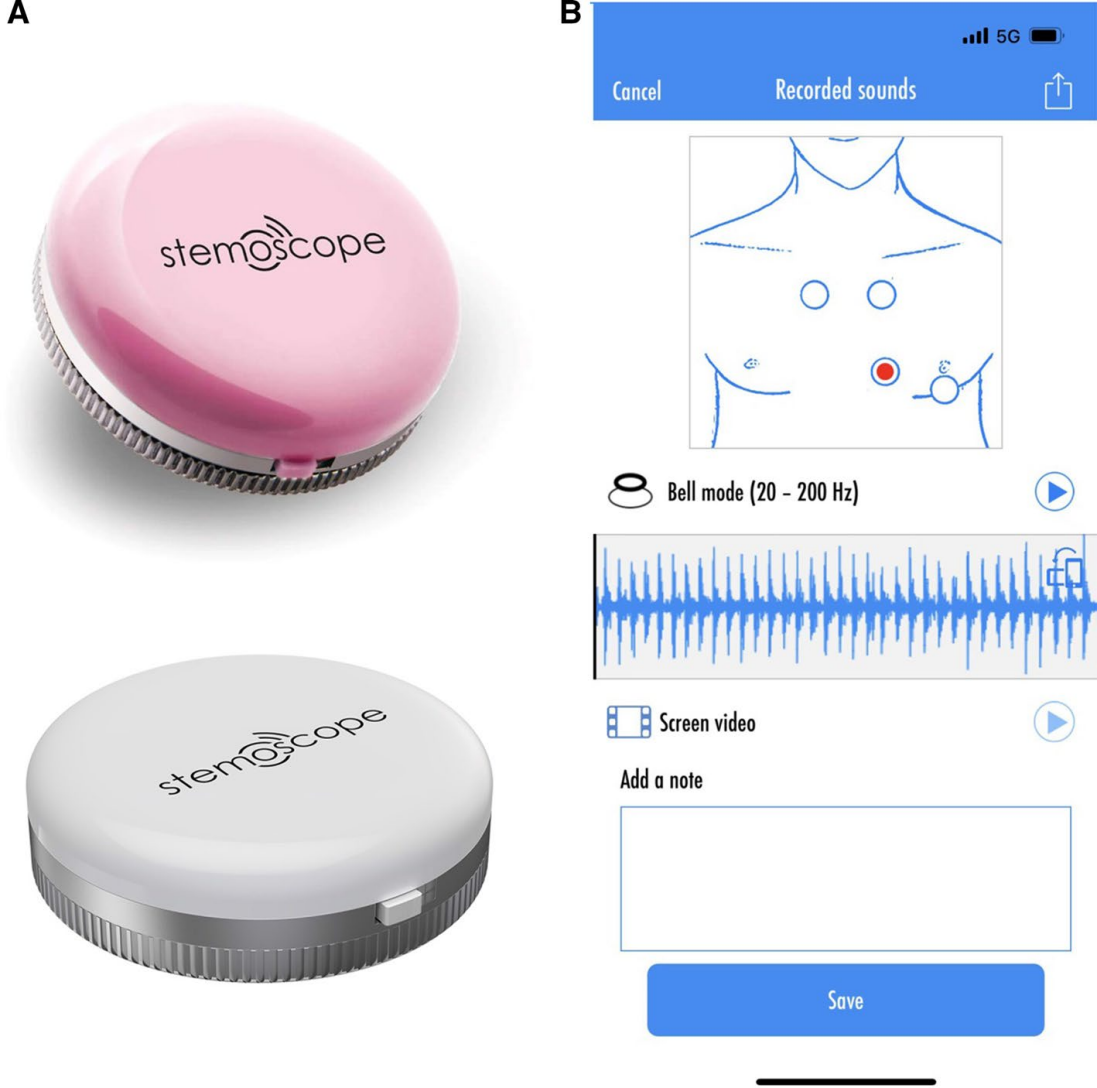
A Stemoscope. **B** Stemoscope app to record heart sounds. The red dot on the screen is tapped to start recording sounds. Then, the investigators can play the recorded sounds. The app provides several options to share recorded sounds, such as via Twitter, Facebook, and WeChat

Three cardiologists with approximately 20 years of experience were asked to perform two auscultations in the same patient group using both a traditional acoustic stethoscope (3M Littman Cardiology III Mechanical Stethoscope, 3M Health Care) and an electronic stethoscope (Stemoscope, Hulu Devices). The Stemoscope consists of a Bluetooth hardware device and a software app (Fig. 4).

Before auscultation, each cardiologist watched a 2-min video introducing the electronic stethoscope and explaining how to use it. They also had 2 weeks to practice using the electronic stethoscope before the study. First, the cardiologists were asked to finish the routine acoustic auscultation in five standard precordial locations (mitral, tricuspid, ERBs, aortic and pulmonary) or with the Stemoscope in a randomized manner. In the electronic in-person auscultation step, heart sounds were also recorded in 15-s intervals for later tele-auscultation use. Sounds were recorded with the patients in sitting or supine positions. The recorded sound files contained annotations detailing the precordial location and the patient’s name and age. Finally, after a month, the grouped recordings for each patient was sent to the same three cardiologists using a secure email sharing function or WeChat for tele-auscultation analysis. During the whole study, the cardiologists were blinded to the previous in-person examination findings, the clinical diagnosis, and the echocardiographic findings. Due to the design of the Stemoscope, the recordings also included an associated phonocardiogram.

Physicians were allowed to listen several times until they were satisfied with the sound quality during one auscultation. There was a time frame of 12 h or less between the completion of the first and second auscultations to keep the results as accurate as possible and to minimize bias. Three cardiologists performed the listening and recording by a standardized pair of AirPods headphones. They were asked to categorize the sounds as normal or abnormal. Abnormal examinations were then further classified as one or more of the following: holosystolic murmur, systolic ejection murmur, diastolic murmur, continuous murmur, click, S2, S3, S4, prosthetic valve sounds, atrial fibrillation, and premature beats. The findings were reported in a standardized form.

The participants underwent an echocardiography check within one month, to which the listeners were blinded. Echocardiography is the gold standard for evaluating a murmur. The findings of the sound recordings were compared against the echocardiographic findings. Finally, a qualitative questionnaire was used to assess the cardiologists’ experience using the Stemoscope system and their attitude toward the future use of tele-auscultation.

Whether murmurs are consistent with echocardiography findings is related to diagnostic accuracy. Generally, if a patient has a systolic murmur in the mitral valve area and echocardiography confirms only mitral regurgitation, then the auscultatory diagnosis is correct; otherwise, it is incorrect. Suppose a patient has systolic murmurs in several valves, and echocardiography confirms only mitral regurgitation (murmurs in other valves may be caused by conduction, which is difficult to identify). In that case, the auscultatory diagnosis is also defined as correct. Suppose a patient has both a systolic and a diastolic murmur in the mitral valve area, but echocardiography confirms only mitral regurgitation. In that case, the auscultatory diagnosis is correct for the systolic murmur but false positive for the diastolic murmur and vice versa. Suppose a patient has only a systolic murmur in the mitral valve area, but echocardiography confirms mitral regurgitation and stenosis. In that case, the auscultatory diagnosis is correct for the systolic murmur, but false negative for the diastolic murmur and vice versa.

### Statistical analysis

Categorical variables were presented as frequency rates and percentages. Continuous variables were presented as means ± standard deviations (SDs). McNemar’s test was used to compare the percent agreement in the following three scenarios: in-person auscultation, tele-auscultation, and echocardiography findings. The kappa value, sensitivity, and specificity were calculated. When comparing the auscultation results with the echocardiography findings, each echocardiography-confirmed lesion was included in the analysis. The analysis aimed to observe if there was an abnormality observed/heard. Systolic and diastolic murmurs were also analyzed separately. Unlike some studies that only compare aortic stenosis or mitral regurgitation murmurs, we compared all murmurs in this study. For this analysis, Table 6 shows the definition for correctly identifying an echocardiography finding by auscultation. We also defined the criteria for combination lesions. Patients with more than one valvular lesion and those with both systolic and diastolic murmurs will be analyzed separately and together according to the different purposes. The significance level was < 0.05.

**Table 6:** Definition for the correct identification of an echocardiography finding by auscultation

	Echocardiography	
	Not observed	Observed
*Stethoscope*		
Not heard	Correct	False-negative
Heard	False-positive	Correct

## Abbreviations

VHD: Valvular heart disease
COVID-19: Coronavirus disease 2019
S1: Heart sound 1
S2: Heart sound 2
S3: Heart sound 3
S4: Heart sound 4
SD: Standard deviation

## Declarations

### Availability of data and materials

All relevant data are contained within the article.
